# Acidic polymers reversibly deactivate phages due to pH changes†

**DOI:** 10.1039/d4lp00202d

**Published:** 2024-08-23

**Authors:** Huba L. Marton, Antonia P. Sagona, Peter Kilbride, Matthew I. Gibson

**Affiliations:** a Department of Chemistry, University of Warwick Coventry CV4 7AL UK matt.gibson@manchester.ac.uk +44 247 652 4112; b Warwick Medical School, University of Warwick Coventry CV4 7AL UK; c School of Life Sciences, University of Warwick Coventry CV4 7AL UK A.Sagona@warwick.ac.uk; d Asymptote, Cytiva, Chivers Way Cambridge CB24 9BZ UK; e Department of Chemistry, University of Manchester Oxford Road Manchester M13 9PL UK; f Manchester Institute of Biotechnology, University of Manchester 131 Princess Street Manchester M1 7DN UK

## Abstract

Bacteriophages are promising as therapeutics and biotechnological tools, but they also present a problem for routine and commercial bacterial cultures, where contamination must be avoided. Poly(carboxylic acids) have been reported to inhibit phages’ ability to infect their bacterial hosts and hence offer an exciting route to discover additives to prevent infection. Their mechanism and limitations have not been explored. Here, we report the role of pH in inactivating phages to determine if the polymers are unique or simply acidic. It is shown that lower pH (=3) triggered by either acidic polymers or similar changes in pH using HCl lead to inhibition. There is no inhibitory activity at higher pHs (in growth media). This was shown across a panel of phages and different molecular weights of commercial and controlled-radical polymerization-derived poly(acrylic acid)s. It is shown that poly(acrylic acid) leads to reversible deactivation of phage, but when the pH is adjusted using HCl alone the phage is irreversibly deactivated. Further experiments using metal binders ruled out ion depletion as the mode of action. These results show that polymeric phage inhibitors may work by unique mechanisms of action and that pH alone cannot explain the observed effects whilst also placing constraints on the practical utility of poly(acrylic acid).

## Abstract 2

The role of acidic polymers in neutralising bacteriophages is investigated.

## Graphical abstract

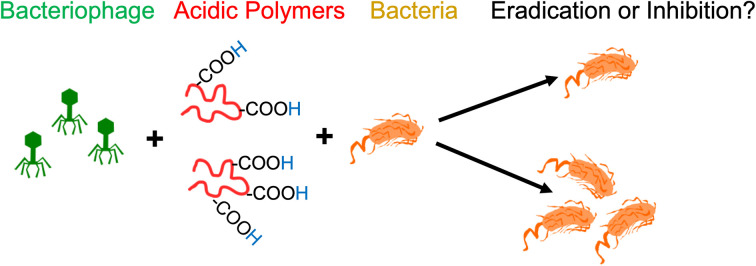

## Introduction

Biosynthetic pathway editing is used in bacteria to produce high-value chemicals and natural products.^1^ The broad uses of bacteria in food production range from dairy products, including cheese and yoghurt, to pickled products, such as sauerkraut, gherkins and olives, using lactic acid bacteria.^1^ Hence, for the use of bacteria in any application area, it is essential to exclude bacteriophages (phages – bacteria selective viruses), a common cause of infection leading to financial and scientific losses. Bacteriophages are amongst the most abundant organisms on earth and are present wherever their hosts are.^2^ Phages have potential as alternatives to antibiotics,^3–5^ for food safety,^6^ veterinary settings,^7^ and biotechnology for ligand selection.^8–10^

Despite their wide biotechnological use, phage contamination in bacterial cultures leads to complete loss of the culture. It requires starting re-culturing from uninfected stocks, carrying significant cost implications for academic and industrial settings. For example, removing phages from raw materials in the food industry is almost impossible, often leading to process collapse.^11–13^ Present-day mitigation tools include good microbiology practices, working under aseptic conditions and vigorous cleaning or autoclaving. However, mitigation is not always successful as phages are robust and can survive in almost every condition.^14^ An alternative option is to engineer bacterial strains intrinsically resistant to phages using gene editing technology, but this is not trivial and may not suit all bacterial hosts.^15^ Re-engineering strains optimised for a particular bio-refinery or changing the refinery's processes is not always easy. Hence, a practical solution would be an anti-phage additive, comparable to how antibiotics are routinely used in mammalian cell cultures to prevent bacterial infection.^16^ Several studies explored the use of phages in treatment^7,17,18^ and ligand screening,^9,10,19^ but very few tools to inhibit them. In contrast, investigations of mammalian viruses led to the discovery of viral inhibitors^20,21^ and the successful re-purposing of existing inhibitors.^22^ The evolutionary phage prevention/reducing system bacteria acquired evolving with their phage predators, relying on protein components, restriction-modification, and CRISPR defences,^23–25^ are not easy to re-purpose as an anti-phage additive for biotechnological processes. Some studies reported phage-inhibiting molecules discovered in *Streptomyces*^26^ and some aminoglycoside antibiotics.^27^ Due to antimicrobial resistance (AMR) concerns, the latter is not desirable for large-scale biotechnological applications.

Sulphated polymers, which mimic heparin sulphate anchors on cell membranes and some modified cyclodextrins, have been recently shown to be broad-spectrum virucides against various human pathogenic viruses.^28,29^ Poly(carboxylic acids) have also been reported to inhibit human viruses.^30–32^ It is well established that polymers can also be deployed as anti-bacterial agents, mimicking cationic host-defence peptides.^33–36^ Mild acidic conditions have also been reported to inhibit mammalian viruses.^37^ Apart from the carboxylic acids, fatty acids and phenolic acids have been reported as mammalian virus inhibitors.^38,39^ We have previously reported that poly(carboxylic acids) can inhibit phage replication.^40^ However, the mode of action is still being determined, and these polymers’ limits and practical utility have yet to be explored.

Here, we report the further investigation of poly(acrylic acid) as a phage inhibitor, focusing on the role of pH. The phage is irreversibly inactivated after exposure to low pH (3) using HCl. In contrast the inactivation is fully reversible when using poly(acrylic acid) to obtain the same pH. This supports the hypothesis that the pH is crucial to inactivation but that the polymers may play a unique role. Depletion of divalent metal ions under the tested conditions was ruled out as a contributor using metal binding macrocycles. This supports the exploration of biomaterials to control phage infection but also highlights that the pH is (currently) the most significant contributor to function.

## Results and discussion

**Fig. 1:**
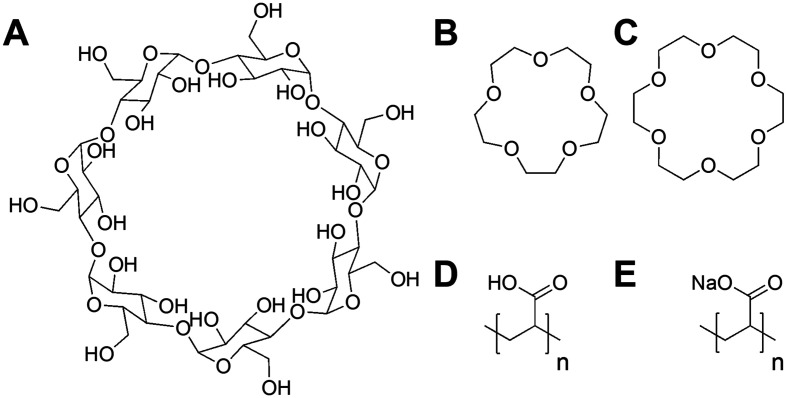
Compounds screened for phage inhibition. (A) β-Cyclodextrin; (B) 15-crown-5 ether; (C) 18-crown-6 ether; (D) RAFT synthesised poly(acrylic acid) acid form (full synthesis details in ESI†); (E) commercial poly(acrylic acid) sodium salt form.

Poly(acrylic acid) was previously reported to inhibit the infectivity of *Escherichia coli* targeting bacteriophage through a virustatic (reversible) mechanism without impacting host biotechnological processes.^40^ To further elucidate the inhibition mechanism and to compare in-house synthesised (using RAFT polymerisation) (full details in ESI†) *versus* commercially available PAA on inhibition, commercial poly(acrylic acid) (5000 g mol^−1^) was tested. The rationale is that other users might buy commercial polymers but these are supplied in both acid and basic forms: a distinction we show here to be crucial. Commercial PAA was purchased as a sodium salt form (Fig. 1), so upon direct dissolution in water, it gives a higher pH value than the same experiment using the acid form (which was synthesised in-house). In addition to the observations of the polyacids, we wanted to rule in/out a mechanism of inhibition for bacteriophages involving the sequestration of divalent ions, such as calcium (Ca^2+^), which are essential for the proliferation of bacteriophages.^41–45^ Calcium ions can increase the rate at which bacteriophages bind to their host,^46^ which may be replaced by other divalent ions such as magnesium (Mg^2+^) or manganese (Mn^2+^),^45^ due to phage adaptability. We hypothesised that the PAA (and poly(methacrylic acid)) could sequester one or more of these divalent ions, leading to the observed inhibitory activity. To address this, beta-cyclodextrins^47–49^ and crown ethers (18-crown-6 and 15-crown-5 ether)^50,51^ were included in our initial screening (Fig. 1) as model metal ion binders. Briefly, each compound was incubated with K1F-GFP and T4 bacteriophages in SM-II buffer with an additive concentration of 10, 15 or 20 mg mL^−1^ overnight to allow interaction between the two. The incubated solution was then added to a culture of *E. coli* EV36 or *E. coli* K-12 (K1F-GFP and T4 phage hosts) grown for 4 hours at 37 °C after seeding. Bacteriophage-infected liquid cultures were grown for 20 hours at 37 °C (24 hours total). Bacterial growth was measured by monitoring the increase in optical density at 600 nm (OD_600_). An increase in OD indicates bacterial growth and hence no phage activity, whilst viable (infectious) phages would inhibit the bacterial growth initially (at *t* = 5 hours) before rebounding during extended culture. The initial low-throughput assay (Fig. S1†) showed that the only bacteriophage inhibitors were the positive controls used (synthetic PAA 32 and PAA 372, numbers corresponding to degrees of polymerisation, full details in Table S1†). The metal chelators had no activity, and neither did the commercial PAA deployed as the sodium salt. Direct dissolution of commercial (Na salt) and home-made (acid form) PAA showed a pH of ∼8 and ∼3, respectively. This observation suggested that pH contributes to phage dissolution and was further explored.

**Fig. 2:**
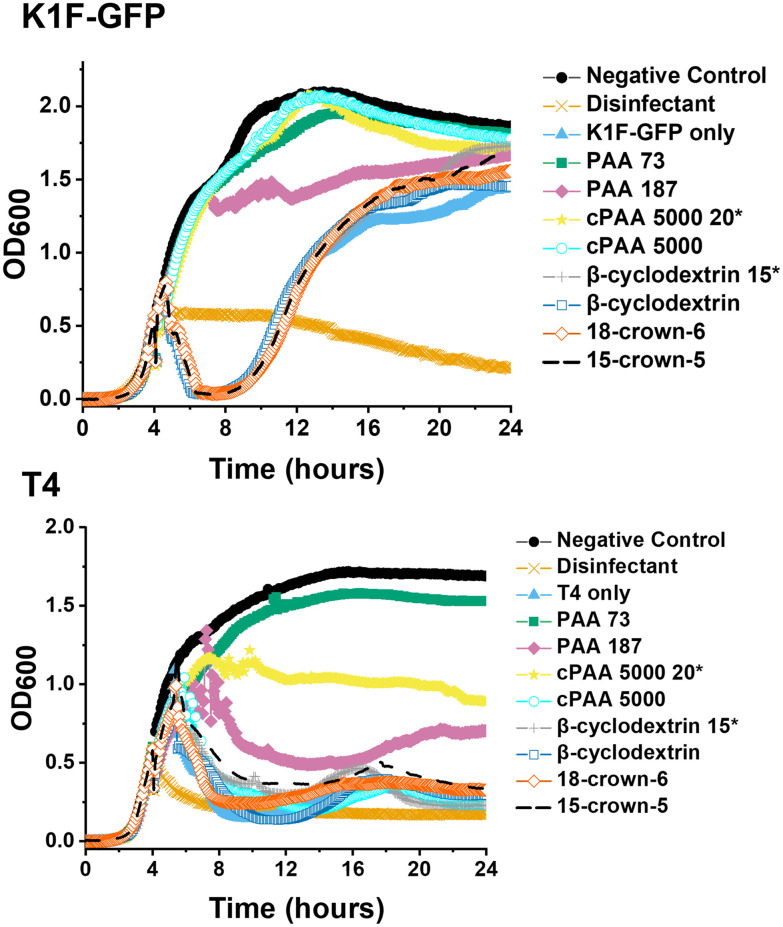
Phage inhibition testing after incubation in SM-II. Growth curves for *E. coli* targeting K1F-GFP and T4 bacteriophages. *E. coli* EV36 was used as the bacteria host for K1F-GFP phages, and *E. coli* K-12 was used as the bacteria host for T4 phages, with a starting concentration of 1 × 10^6^ CFU mL^−1^. The commercial PAA (cPAA 5000) samples were acidified to pH 3, whilst cyclodextrin, crown ethers and in-house synthesised PAA 73 and PAA 187 were used directly in the media. Unless marked by an asterisk (20* or 15*), the additive concentration was 10 mg mL^−1^ before incubating phages for 24 hours and adding them to host cultures. K1F-GFP and T4 controls refer to non-additive-containing phage aliquots. LB media was used as a negative control, and 1% Chemgene disinfectant was used as a positive control. The growth curves represent one biological and two technical repeats.

Commercial PAA aliquots were acidified with dilute HCl to pH 3. The final commercial PAA concentration of 10 mg mL^−1^ and 20 mg mL^−1^ matched the previously reported MIC of synthetic PAA for K1F-GFP and T4, respectively.^40^ Post-acidification, the same screening phage inhibition assay as in a previous report was used,^40^ including all tested compounds for comparative purposes (Fig. 2).

The data in Fig. 2 demonstrated that acidification of the commercial (sodium salt) PAA to pH 3 re-activated its phage inhibitory ability at both 20 mg mL^−1^ and 10 mg mL^−1^ for K1F-GFP with a notably increased potency at 20 mg mL^−1^ for T4, matching the performance of the in-house synthesised (acid form) PAA. This result confirms the hypothesis that poly(acids) phage-inhibitory activity is linked to pH, and when used directly as the sodium salt (as in the commercial sample), no activity is seen.

**Fig. 3:**
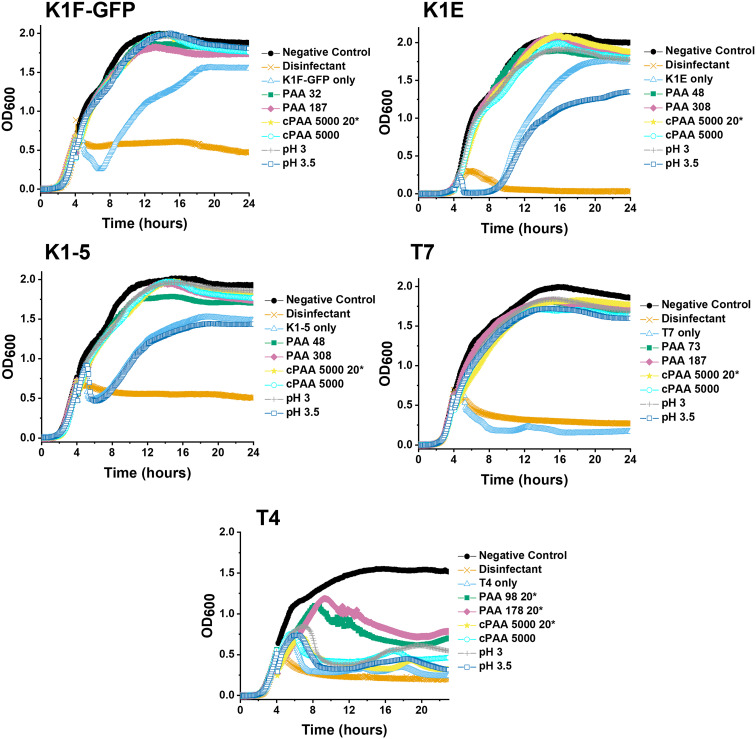
Higher-throughput testing for phage inhibition. 24-hour growth curves of each bacteriophage. *E. coli* EV36 was used as the bacteria host for K1F-GFP, K1E, and K1-5 phages, whilst *E. coli* K-12 was used as the bacteria host for T7 and T4 phages, with a starting concentration of 1 × 10^6^ CFU mL^−1^. Commercial PAA (cPAA) was acidified to pH 3. Polymer concentration was 10 mg mL^−1^ unless marked by an asterisk (20* to indicate 20 mg mL^−1^) before incubating phages for 24 hours and adding them to 4-hour-grown host cultures. Except for T4, all phages were incubated in 10 mg mL^−1^ PAA 98 and PAA 178. ‘Phage only’ samples correspond to non-additive-containing phage aliquots. LB media = negative control, 1% v/v Chemgene = positive control. The growth curve assay represents three biological and three technical repeats.

Guided by the above, a higher-throughput assay was carried out for five different *E. coli* targeting bacteriophages (K1F-GFP, K1E, K1-5, T7, and T4) to investigate our initial observations further. Commercial PAA sodium salt 5000 g mol^−1^ (cPAA) synthetic PAA 98, and PAA 178 (number represents the degree of polymerisation) were used at 10 mg mL^−1^ for all phages, except T4, where 20 mg mL^−1^ is needed to reach the MIC value and to avoid false negatives. The commercial PAAs were acidified to pH 3 before testing, and controls of acidified SM-II buffers to pH 3 and 3.5 were prepared to test the effects of low pH without PAA (Fig. 3). Spot tests (solid phase growth assay) for phage were also performed to quantify the impact further, using 1–6 10-fold dilution segmented plates of the corresponding *E. coli* host lawn (Fig. S2†). The high-throughput assay revealed phage infectivity inhibition for all the (pH = 3) polymer samples and the acidified SM-II buffer. These results were confirmed using the spot test data by the absence of phage plaques on the plates. This critical observation suggested low pH was vital to the phage inhibition mechanism.

The variable pH inhibition was investigated after confirming that acidity (low pH) assisted phage inhibition for the five bacteriophages. Before the growth curve assay, three separate series of dose–response conditions were performed: acidification of the SM-II buffer without PAA to pH 3, commercial PAA to pH 3 and NaOH basification of the synthetic PAA to pH 5.5. This dose–response showed that acidification of the commercial PAA (decreasing pH) leads to an ‘activation’ of the phage inhibition (Fig. S3†). The reverse of this trend also held, with basification (increasing pH) leading to the ‘deactivation’ of the otherwise potent 10 mg mL^−1^ synthetic PAA, which enabled bacterial growth, *i.e.* lack of phage inhibition (Fig. S3†). The minimum inhibitory pH values from the dose–response curves are summarised in Table 1. In all three conditions, a pH value below 3 inhibited all three tested phages (K1F-GFP, K1-5, and T7), regardless of the starting pH, before increasing or decreasing this.

**Table 1:** Minimum inhibitory pH from the dose–response solution-phase screening

Condition	Phage and minimum inhibitory pH		
	K1F-GFP	K1-5	T7
Acidified SM-II	<3	3	3
Acidified cPAA^a^	3	3	3
Basified sPAA^b^	3.5	3.5	3.5

a cPAA = commercial poly(acrylic acid) (MW = 5000).

b sPAA = synthetic poly(acrylic acid) PAA 153.

The data above showed that an acidic environment was a vital aspect of the *E. coli* targeting phage inhibition mechanism with or without the presence of poly(acrylic acid). Low pH-assisted inhibition of mammalian viruses has been shown, with Janiga *et al.* reporting mild acidic pH inhibition of Herpes simplex virus (HSV).^37^ Damonte reported the inhibition of Junin virus (JUNV), an arenavirus, by fatty acids, including capric, lauric, myristic, palmitic, and stearic acids.^38^ Mechanistic studies concluded that lauric acid inhibited a late maturation stage in the replication cycle of JUNV rather than the inactivation of virions. In contrast, varied chain-length fatty acids had negligible anti-JUNV activity.^38^ Silva Jr. reported the *in vitro* inhibition of canine distemper virus (CDV) by phenolic acids (*cis*-cinnamic acid, *trans*-cinnamic acid, and ferulic acid).^39^ Elrod reported the inhibitory activity of medium-chain fatty acids, including caprylic, capric, and lauric acids, on the African swine fever virus (ASFv).^52^

**Fig. 4:**
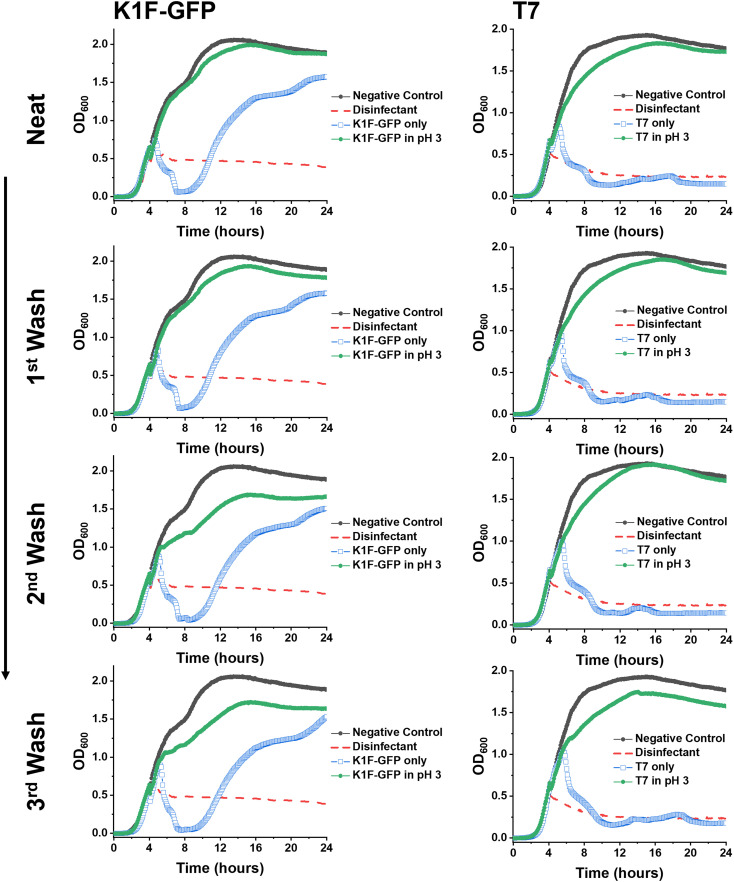
Virustatic *versus* virucidal testing of low pH on bacteriophages. Growth curves of K1F-GFP and T7 bacteriophages after three washing steps (1 : 10 dilution in SM-II phage buffer). *E. coli* EV36 was the bacteria host for K1F-GFP phages, and *E. coli* K-12 (MG1655 cells) was the bacteria host for T7 phages, with a starting concentration of 1 × 10^6^ CFU mL^−1^. The samples were washed (increasing the pH) before adding the aliquots to the pre-log host cultures. ‘Phage only’ samples correspond to non-additive containing bacteriophage aliquots (matching the concentration of the acidified (pH 3) sample of the corresponding washing step). LB media was used as a negative control, and 1% Chemgene disinfectant was used as a positive control. The growth curves represent a single biological and four technical repeats.

Synthetic PAA was previously reported to inhibit phage activity through a virustatic (reversible) mechanism.^40^ The low pH high-throughput assay and the pH dose–response data both suggested that below pH 3, the bacteriophages were inhibited, with and without the presence of PAA. Based on this, the next question was whether the mechanism of inhibition was simply due to a low pH or whether acidic PAA inhibited the phages differently from acidity alone. Hence, an experiment was devised to determine if the acidic (low pH) conditions permanently (*i.e.*, virucidal) or transiently (*i.e.*, virustatic) inhibit the phages, so that the data was comparable to washing out the polymers. Briefly, K1F-GFP and T7 phages were incubated with acidified SM-II to pH 3 (minimum inhibitory pH, so no subsequent infection would be expected) for 24 hours. After incubation, each acidified phage solution was diluted (washed out), which increased the pH above 3 (the inhibition threshold) before being added to the *E. coli* host. Up to three 10-fold dilutions, the pH 3 exposed bacteriophages were still unable to eradicate the bacteria, which confirmed a virucidal (irreversible) mechanism of inhibition at low pH conditions (Fig. 4). This was confirmed by the phage infectivity in the untreated phage controls applied at equal PFU per mL to account for the dilution. These contrast when PAA is used against the phages using the same experimental set up, which shows fully reversibly inhibitory activity.^40^

Taking into account the impact of pH on the inhibition of bacteriophages, two pH tracking assays were devised to measure the changes in pH at each stage of the growth curve assay and pH wash assay from incubated aliquot to addition to LB media. The difference between synthetic PAA and synthetic poly(methacrylic acid) (PMA) was compared in the first pH tracking assay, as PMA was reported as a weak phage inhibitor.^40^ Briefly, the pH was measured where a corresponding phage-free SM-II buffer replaced the phage-containing volume to mimic the former. The tracking assay showed that this led to an increase in the pH of PMA across dilutions (Table S1†) as would be expected. At 10 mg mL^−1^, the difference in pH between PMA/PAA was ∼0.3, which may explain the difference in inhibition potency due to the pKa differences between PAA and PMA. Diluting the PAA in LB media led to similar pHs compared to the pH solution alone, confirming that the final bacterial exposure conditions were identical (Table S2†).

**Fig. 5:**
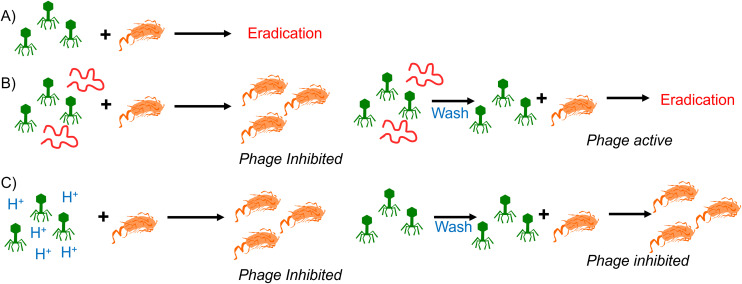
Summary of phage inhibition observations. (A) Typical phage eradication; (B) reversible poly(acrylic acid) (PAA) (red chain) phage inhibition upon washing; (C) irreversible acidity (blue protons) phage inhibition upon washing.

This data confirms that the decrease in pH is essential for the anti-phage activity of poly(carboxylic acids) and that simply lowering pH is an effective tool to neutralise phage contamination. However, it also showed that phages that had been neutralised by poly(carboxylic acids) recovered their activity when the pH was returned to neutral. The reasons for this remain unclear, but an electrostatic interaction between the polymer and the phage surface could lead to a protective effect against the drop in pH and hence the poly(acrylic acid) serve both as source of low pH but mitigate some of the impact. Fig. 5 summarizes our observations of polymers at the same pH, reversibly inactivating phage, but the same pH without polymer is irreversible deactivation. It shows that polymers have distinct mechanisms and might give an opportunity to discover materials that are selective and more active for phage eradication.

## Conclusions

Here, we report a comparison of the inhibition of bacteriophages by polymeric acids to the inhibition caused by simple modulation of pH. Poly(acrylic acid) in its acid form was first shown to be more active than its sodium salt, consistent with the pH playing a pivotal role. Acidification of commercial poly(acrylic acid) sodium salt to pH 3 re-activated the phage inhibitory mechanism. It was observed that if a phage solution was acidified to pH 3 (using HCl), phage was irreversibly deactivated, such that when applied to a bacterial culture (with the pH recovered back to that of the growth media), the bacteria could grow as expected. In contrast, when the poly(acrylic acid)s were cycled from pH3 back to the growth media, the phage activity was recovered (*i.e.* bacteria growth inhibited), confirming that poly(carboxylic acid)s have a fully reversible mode of action that is linked to pH, but still distinct. As a potential mode of action, the sequestration of divalent metal ions by the poly(acids) was ruled out through testing of metal binding macrocycles, which showed no impact on phage function under the conditions tested. These results show that polymeric phage inhibitors are potent but function by a mechanism that includes lowering the pH and hence cannot simply be integrated into bacterial cultures unless the bacteria tolerate low pH. However, the reversible nature of the inactivation suggests a unique mechanism that might be exploitable in next-generation polymeric phage inhibitors and will form the basis of future studies.

## Data access

Background data is available in the ESI.†

## Conflicts of interest

MIG, APD and HLM are named inventors on a patent application relating to this work.

## Supplementary Material

LP-002-D4LP00202D-s001
