# Supplementary material for: Acidic polymers reversibly deactivate phages due to pH changes
Source: RSC Appl Polym. 2024 Aug 23;2(6):1082–90. doi: 10.1039/d4lp00202d (PMC11342163; doi:10.1039/d4lp00202d)
Supplement: LP-002-D4LP00202D-s001 [file LP-002-D4LP00202D-s001.pdf]

Supplementary Information (SI) for RSC Applied Polymers.
This journal is © The Royal Society of Chemistry 2024

Supporting Information

Acidic Polymers Reversibility Deactivate Phages due to pH Changes

Huba L. Marton,[a] Antonia P. Sagona,[c*] Peter Kilbride,[d] and Matthew I. Gibson[a,b,e,f *]

a) Department of Chemistry, University of Warwick, Coventry, CV4 7AL, United Kingdom

b) Warwick Medical School, University of Warwick, Coventry, CV4 7AL, United Kingdom

c) School of Life Sciences, University of Warwick, Coventry, CV4 7AL, United Kingdom

d) Asymptote, Cytiva, Chivers Way, Cambridge, CB24 9BZ

e) Department of Chemistry, University of Manchester, Oxford Road, Manchester, M13 9PL, United Kingdom

f) Manchester Institute of Biotechnology, University of Manchester, 131 Princess Street, Manchester, M1 7DN, United Kingdom

CORRESPONDING AUTHOR DETAILS

*Fax: +44 247 652 4112. E-mail: matt.gibson@manchester.ac.uk and A.Sagona@warwick.ac.uk

Experimental Section

Materials.

All the materials (reagents and media) were used as supplied unless stated otherwise. 1-Dodecanethiol (≥98%), 2- bromo-2-methylpropionic acid (98%), carbon disulfide (anhydrous, ≥99%), tripotassium phosphate (≥98%), acetone (reagent grade), hydrochloric acid (reagent grade), dichloromethane (≥99.8%), N,N-dimethylformamide (99%, DMF), diethyl ether (≥ 99.7%), (n-hexane (reagent grade), petroleum ether (boiling point 40-60 °C), glacial acetic acid (≥ 99.7%), silica gel, 4,4'-azidobis(4-cyanovaleric acid) (≥75%, ACVA), acrylic acid (99%, AA), methacrylic acid (99%, MA), 2-cyano-2-propyl dodecyl trithiocarbonate (97%), 4-cyano-4-(phenylcarbonothioylthio)pentanoic acid, methanol (≥ 99.9%), chloroform-d_1 (99.8%), methanol-d_4 (≥99.8%), deuterium oxide (99.9%), agarose, lysogeny broth (LB), poly(acrylic acid) (PAA) (Mw 5000), b-cyclodextrin (≥97%), 1,4,7,10,13,16-hexaoxacyclooctadecane (18-crown-6 ether) (≥98%), 1,4,7,10,13-pentaoxacyclopentadecane (15-crown-5 ether) (≥98%) and sodium hydroxide (≥97%) were all purchased from Sigma-Aldrich (Merck). Caesium chloride, magnesium sulfate heptahydrate, sodium chloride and poly(ethylene glycol) (PEG) (Mn 8,000) were purchased from Thermo Fisher Scientific. Glycerol (≥99%) was purchased from Scientific Laboratory Supplies (SLS). Tris-HCl (24.2 $g.L^{-1}$ Tris, 80 $g.L^{-1}$ NaCl), kanamycin (50 $\mu g.mL^{-1}$) and ampicillin (100 $\mu g.mL^{-1}$) were prepared by the media preparation facility of the School of Life Sciences at the University of Warwick. SM-I buffer (1 M NaCl, 8 mM $MgSO_4.7H_2O$, 22.5 mM Tris-HCl pH 7.5) and SM-II buffer (100 mM NaCl, 8 mM $MgSO_4.7H_2O$, 22.5 mM Tris-HCl pH 7.5) were prepared at the University of Warwick (in house).

Physical and Analytical Methods

NMR spectroscopy.

Proton (^{1}H-NMR) nuclear magnetic resonance spectra were recorded at 300 MHz or 400 MHz on a Bruker DPX-300 or DPX-400 spectrometer, respectively, with chloroform-d ($CDCl_3$), deuterium oxide (D_2O) and methanol-d4 (CD_3OD) as the solvents. Chemical shifts of protons are reported as δ in parts per million (ppm) and are relative to tetramethylsilane (TMS) at δ = 0 ppm when using $CDCl_3$ or solvent residual peak (CH_3OH, δ = 3.31 ppm) for CD_3OD, (H_2O, δ = 4.78 ppm).

Fourier-Transformed infrared (FT-IR) spectroscopy.

Fourier-transform infrared (FT-IR) spectroscopy measurements of each synthesised compound were carried out in the 650 to 4000 cm-1 range using a Cary 630 FT-IR spectrometer (Agilent).

Size exclusion chromatography (SEC) in DMF.

For each polymer synthesised, size exclusion chromatography (SEC) analysis was performed on an Agilent Infinity II MDS instrument equipped with differential refractive index (DRI), viscometry (VS), dual-angle light scatter (LS) and variable wavelength UV detectors. The system had 2 × PLgel Mixed D columns (300 × 7.5 mm) and a PLgel 5 µm guard column. The mobile phase used was DMF (HPLC grade) containing 5 mM NH_4BF_4 at a flow rate of 1.0 mL.min^{-1} at 50 ^{o}C. Poly(methyl methacrylate) (PMMA) standards (Agilent EasyVials) were used for calibration between 955,000 – 550 g.mol^{-1}. Analyte samples were filtered through a nylon membrane with 0.22 μm pore size before injection. Number average molecular weights (M_n), average molecular weights (M_w) and dispersities ($Đ_M$ = M_w/M_n) were determined by conventional calibration and universal calibration using Agilent GPC (gel permeation chromatography)/SEC software.

Optical density measurements.

For the optical density measurements at 600 nm (OD_{600}) of bacterial cultures before each growth curve described below, a Fisher Scientific portable cell density meter, model 40, was used. The optical density of the bacterial cultures during the growth curve assay described below was measured using a FLUOstar Omega microplate reader (BMG Labtech), taking OD_{600} measurements every five minutes over 24 hours.

pH measurements.

To prepare the acidified commercial poly(acrylic acid) containing SM-II buffers and subsequent pH tracking experiments, pH measurements were performed on a BASIC pH Meter (Denver Instrument, Switzerland), calibrating the instrument before each set of readings with pH adjusted by addition of $HCl_{(aq)}$ or $NaOH_{(aq)}$.

Polymer Synthesis. The polymers used featured in a previous report. [2] For clarity the full synthesis details are included here to aid the reader but it is important to note the reason for the identical characterization data is for this reason.

Synthesis of *2-(dodecylthiocarbonothioylthio)-2-methyl propionic acid (DMP)*

2-(dodecylthiocarbonothioylthio)-2-methyl propionic acid (DMP) was synthesised using a previous procedure.[1] 1-Dodecane thiol (2.00 g, 9.88 mmol) was slowly added to stirring K_3PO_4 (2.10 g, 9.89 mmol) in acetone (30 mL) at RTP (room temperature and pressure), leaving the new mixture stirring for 25 minutes until a white suspension formed. After adding carbon disulphide (2.05 g, 26.93 mmol), the mixture was stirred for 10 minutes to form a yellow solution. 2-bromo-2-methyl-propionic acid (1.5 g, 8.98 mmol) was added, the solution was left stirring for 16 hours, and the solvent was removed under a vacuum. The crude product was dissolved in 1M HCl (100 mL) and extracted with DCM (2 × 100 mL). Afterwards, the organic layer was washed with water (200 mL) and brine (200 mL), dried with $MgSO_4$ and filtered under gravity. The solvent from the filtrate was removed under vacuum. Finally, the crude product was purified through a silica column (40-60 PET: DCM: glacial acetic acid 75:24:1)

and recrystallised in n-hexane to give a yellow solid (32%). ^{1}H NMR (300 MHz, $CDCl_3$) δ = 3.30 (2H,t, SCH_2**CH_2**), 1.75 (6H, s, C**(CH_3)$_2$**), 1.69 (2H, qn, S**CH_2**), 1.46 - 1.22 (18H, m,(**CH_2**)$_9$$CH_3$), 0.93 - 0.87 (3H, m, **CH_3**). m/z calculated as 364.16; found for ESI $[M+H]^+$ 365.1 and $[M+Na]^+$ 387.1. FTIR (cm^{-1}) – 2956, 2917 & 2848 (methyl and methylene), 1702 (ester C=O), 1459, 1437 & 1413 (methyl and methylene), 1280 (C**(CH_3)$_2$**), 1064 (S-C(S)-S).

Poly(acrylic acid) and poly(methacrylic acid) were previously synthesised in the initial screening for bacteriophage inhibition,[2] and repeated in the current work for comparative purposes as inhibitor controls.

Synthesis of Poly(acrylic acid) (PAA)

As a representative example, acrylic acid (1.98 g, 27.46 mmol, 100 eq), previously synthesised DMP (0.1 g, 0.27 mmol, 1 eq), 4,4'-azidobis(4-cyanovaleric acid) (ACVA) (0.015 g, 0.055 mmol, 0.2 eq) and methanol (10.5 mL) were added to a glass reaction vial. The stirrer bar was added, the vial sealed, and the solution degassed with nitrogen for 20 minutes. A small sample was taken to determine conversion by NMR, and the reaction was left stirring at 60 ºC overnight. The next day, the reaction was quenched by submerging the glass vial in liquid nitrogen and exposing the solution to air. The crude polymer was precipitated from diethyl ether and dried under vacuum. Finally, the resulting polymer product was analysed by ^{1}H NMR and SEC. Representative characterization data for PAA_{100}: ^{1}H NMR (400 MHz, CD_3OD): δ 3.45 – 3.38 (4H, m, C(S)S**(CH_2)$_2$**CH_2CH_2), 2.78 – 2.66 (6H, m, COOHC**(CH_3)$_2$**), 2.64 – 2.25 (1H, m, S**CH**(COOH)CH_2) , 2.22 – 1.48 (2H, m, SCH(COOH)**CH_2**) 1.46 – 1.28 (18H, m, CH_3**(CH_2)$_9$**CH_2), 0.96 – 0.89 (3H, t, **CH_3**(CH_2)$_{11}$SCS). M_n^{SEC} (DMF) = 11000 g.mol^{-1}, $Đ_M$ = 1.28.

An alternative chain transfer agent used was 2-Cyano-2-propyl dodecyl trithiocarbonate.

Synthesis of Poly(methacrylic acid) (PMA)

Methacrylic acid (0.46 g, 5.36 mmol, 50 eq), 4-cyano-4-(phenylcarbonothioylthio) pentanoic acid (0.03 g, 0.108 mmol, 1 eq), 4,4'-azidobis(4-cyanovaleric acid) (0.006 g, 0.021 mmol, 0.2 eq) and methanol (2.5 mL) were added to a glass reaction vial. A stirrer bar was added, the vial was sealed, and the solution was purged with nitrogen for 20 minutes. A small sample was taken to determine conversion (NMR), and the reaction was left stirring at 60 ºC overnight. The next day, the reaction was quenched in liquid nitrogen and exposed to air. The crude polymer was precipitated from diethyl ether and dried under vacuum. 1H NMR and SEC analysed the final product. Representative characterization data for PMA_{50}: ^1H NMR (400 MHz, CD_3OD): δ 7.93 – 7.34 (5H, m, Ar), 3.78 – 3.58 (3H, m, C(**CH_3**)$CNCH_2CH_2$), 2.29 – 1.74 (2H, m, SCSC(CH_3)(COOH)**CH_2**), 1.72 – 1.47 (2H, m, COOH**CH_2**CH_2)), 1.46 – 1.29 (2H, m, $COOHCH_2$**CH_2**), 1.15 – 1.00 (3H, m, SCSC(**CH_3**)(COOH)CH_2). M_n^{SEC} (DMF) = 13300 $g.mol^{-1}$, $Đ_M$ = 1.11.

Table S1. Polymer characterisation.Poly(acrylic acids) and poly(methacrylic acid) were previously synthesised for another report but the characteristics are repeated here for clarity [2].

Polymer Code	[M]:[CTA] (-)	$Mn_{,SEC}$ ($g.mol^{-1}$)	$Đ^a$ (-)	DP^b (-)
**PAA 73**	25	5300	1.18	73
**PAA 32**	50	2274	1.10	32
**PAA 98**	50	7101	1.18	98
**PAA 153**	100	11000	1.28	153
**PAA 178**	200	12841	1.43	178
**PAA 187**	200	13500	1.26	187
**PAA 372**	500	26800	1.34	372
**PMA 155**	50	13300	1.11	155

a) Dispersity, M_w/M_n; b) number average degree of polymerisation from SEC data.

Biological Methods

Viral enrichment – propagation of bacteriophages.

During the propagation of the panel of bacteriophage isolates used, *Escherichia coli* EV36 host for K1F-GFP, K1E and K1-5 and *E. coli* AB1157 host for T7 and T4 phages were grown overnight in lysogeny broth (LB) (Lennox – 10 $g.L^{-1}$ tryptone, 5 $g.L^{-1}$ yeast extract, 5 $g.L^{-1}$ NaCl) at 37 °C and 130 rpm. *E. coli* AB1157 was only used to propagate T7 and T4 phages, whereas the host for the 'T' phages was *E. coli* K-12 (MG1655 cells). The following day, 1 mL of the overnight liquid culture was used to inoculate 50 mL of fresh LB for each phage separately. This newly inoculated sample was then incubated at 37 °C and 130 rpm until an OD_{600} (optical density at 600 nm) of 0.3 was reached. At this point, 100 - 300 µL of bacteriophage stock (depending on the previous stock concentration) was added to each corresponding flask and the samples were further incubated for 4 hours. The bacterial debris of *E. coli* EV36 and AB1157 hosts were pelleted by centrifugation at 3220 g for 10 min before passing the supernatants through a 0.2 µm pore size membrane filter. Afterwards, all five prepared bacteriophage stocks in LB were stored at 4 °C.

Caesium chloride purification of bacteriophages.

For the purification of each phage, the previously described propagation assay was scaled up to 250 mL per phage sample by transferring the supernatant (bacteriophage stock) into LB media. Sodium chloride was then added to each phage sample to achieve a final concentration of 1 M. After 1-hour incubation on ice, each phage sample was centrifuged at 3220 g, and the supernatant was filtered through at 0.2 µm pore size membrane before the addition of PEG 8000 to a final concentration of 10% w/v. The phage samples were left overnight at 4 °C before further centrifugation at 25 000 g for one hour. Phage pellets were resuspended in 6-7 mL SM buffer I (gentle washing of the centrifuge container) and passed through a 0.2 µm pore size membrane before undergoing concentration and purification in a CsCl gradient for 20 hours at

150,000 g and 4 °C. Following the centrifugation, phages were concentrated into a band. The band was syringe extracted by piercing and dialysed once in SM-I and twice in SM-II buffer to gradually replace the CsCl with NaCl. Each purified phage stock was then stored at 4 °C.

***Escherichia coli* infection growth curves – low-throughput screening.**

For low- and high-throughput screening, the E. coli EV36 and K-12 cultures were grown separately in a FLUOstar Omega microplate reader at 37 °C, taking optical density measurements (OD_{600} or Abs_{600}) every five minutes over 24 hours. Following on from the synthetic poly(acrylic acid) inhibition work,[2] commercial poly(acrylic acid) (Mw 5000) was tested at 20 $mg.mL^{-1}$ and 10 $mg.mL^{-1}$ inhibitor concentration, with additional additives including β-cyclodextrin (15 and 10 $mg.mL^{-1}$), 18-crown-6 ether (10 $mg.mL^{-1}$) and 15-crown-5 (10 $mg.mL^{-1}$). Before the growth curve assays, bacteriophages were incubated in additives (10, 15 or 20 $mg.mL^{-1}$) dissolved in SM-II buffers overnight, with a starting concentration of at least 1×10^6 $PFU.mL^{-1}$ (plaque-forming units). Bacteria hosts with a final concentration of 1×10^6 $CFU.mL^{-1}$ (colony-forming units) (OD_{600} 0.001) were transferred to a 96-well plate and grown for four hours at 37 °C with shaking to reach the start of the log phase. During the log phase, the overnight additive-incubated phage aliquots were added to corresponding wells of the 96-well plate, including 1% v/v Chemgene™ (HLD_4L) laboratory disinfectant as a positive control. SM-II buffer incubated phages were used as additional controls, and LB media was used as a negative control. All bacteria + phage samples were grown shaking in lysogeny broth (LB) media in a total volume of 200 µL, including LB blank wells. Data was acquired and blank-corrected using the MARS analysis software (Version 4.10).

During the preliminary low-throughput assays, the commercial PAA samples (poly(acrylic acid) sodium salt form) were tested against the synthetic PAA, un-acidified. The rest of the additives were not altered throughout the preliminary testing.

Following this, the cPAA 5000 was acidified to pH 3, using dilute hydrochloric acid (exact

concentration not measured) to ensure the SM-II buffer composition remained the same. This acidification was performed to lower the pH of the commercial PAA (sodium salt form) to match the synthetic PAA.

High-throughput acidified commercial poly(acrylic acid) and low-pH screening.

After the low-throughput screening of the commercial poly(acrylic acid) with and without acidification, high-throughput screening was conducted for the five *E. coli* bacteriophages, K1F-GFP, K1E, K1-5, T7 and T4. Acidified SM-II buffer controls were included at pH 3 and 3.5 to match the pH of the synthetic and commercial poly(acrylic acid) evaluated. The previously described 24-hour growth curve assay was also implemented for the high-throughput screening. Before adding them to the growing host liquid culture, the K1F-GFP, K1-5, T7 and T4 bacteriophages were incubated in SM-II buffer, acidified SM-II (pH 3 and 3.5), commercial and synthetic PAA (20 and 10 $mg.mL^{-1}$) with a final concentration of ~ 1 × 10^6 $PFU.mL^{-1}$. For K1E, a final concentration of ~ 1 × 10^7 $PFU.mL^{-1}$ (10 µL phage + 90 µL additive in buffer) was used.

A lysogeny broth agar (LBA) plate spot test was conducted for each phage screened by serially diluting each 24-hour incubated phage aliquot 10-fold six times. After dilution, 20 µL of each dilution was added to a 1-6 segmented plate to a lawn of growing corresponding *E. coli* EV36 or K-12 host in top-agar (0.7% agarose LBA). This spot test replaced the plaque assay for each bacteriophage tested,[3] As previously shown, inhibition was caused by a lack of plaques (clearance due to bacterial eradication by phage replication) forming on LBA.[2] The LBA plates of each phage sample were photographed as one biological repeat. Growth curve data was collected and blank-corrected on the MARS analysis software (Version 4.10), and each assay was carried out using biological and technical triplicates.

Phage-infected *E. coli* pH dose-response growth curves.

From the high-throughput testing, the acidified SM-II buffer also gave a 'hit' in the inhibitor search. Hence, the subsequent assays evaluated how low pH can inhibit bacteriophage infectivity. SM-II phage buffer aliquots with and without commercial poly(acrylic acid) (10 mg.mL^{-1}) were acidified from pH 8 to 3 using dilute hydrochloric acid. In contrast, the synthetic poly(acrylic acid) containing buffer was, in turn, basified up to pH 4.5 to 'inactivate' the inhibiting effect. NaOH (42.5 mg.mL-1) solution was used for each basification step. In this assay, *E. coli* bacteriophages K1F-GFP, K1-5 and T7 were used, omitting K1E and T4 due to the much lower stock concentration of the lysate, which no longer matched the assay's scope. Each bacteriophage was incubated in buffers of varied conditions (PAA/no PAA) and pH range with a final concentration of ~ 1×10^6 PFU.mL^{-1} (20 µL phage + 180 µL additive in buffer) for 24 hours. After this step, the same method was resumed as the previously described growth curve assay. Data was acquired and blank-corrected on the MARS analysis software (Version 4.10), and each growth curve was conducted in biological singlets and technical triplicates.

Bacteriophage Virustatic- versus Virucidal- assay for low pH.

A modified growth curve assay was conducted to determine if 'washing out' the acidity (pH ≤ 3) in the SM-II could recover the phage infectivity. Briefly, the bacteriophages were incubated in pH 3 (acidified) SM-II buffer for 24 hours at a total volume of 200 µL at 4 °C, with a final concentration of ~ 1×10^{16} PFU.mL^{-1}. K1F-GFP and T7 were chosen as phage representatives with different *E. coli* strain hosts. Host cultures for K1F-GFP (*E. coli* EV36) and T7 (*E. coli* K-12) were grown from a starting concentration of 1×10^6 CFU.mL^{-1} for 4 hours at 37°C to reach the log phase. To assess any loss of inhibition, 10-fold serial dilutions (washes) were performed three times before adding the phages to corresponding host wells at the pre-log phase. Chemgene disinfectant (1% v/v) was added as positive control. For each serial dilution, a bacteriophage control was included with a matching phage titre (PFU.mL^{-1}) to exclude any variation in infectivity due to the reduced number of phages in the culture.

pH change tracking assay.

The final assay carried out was a pH change tracking assay. As the name suggests, the aliquots' pH without adding bacteriophages was measured using two variations of this assay.

In the first variation, synthetic PAA and poly(methacrylic acid) (PMA), previously reported as a weak phage inhibitor,[2] pH was compared at each step of the growth curve assay. The pH was measured from the 20 mg.mL^{-1} PAA/PMA stocks, to mix the aliquot with the LB media to mimic the addition of the 'phages' at the 4-hour pre-exponential growing cultures. SM-II buffer without phage was used as a substitute for the phage-containing SM-II, using the same volume at all corresponding steps to account for this volume difference and the effects it may have had on the pH changes. Hence, 'pseudo' phage mixtures were made (no the addition of phages). Pure lysogeny broth was used as a control for this assay to determine, at the final step, how the pH of the LB media would decrease at the time of interaction between phage and host.

For the second variation of the pH change tracking assay, PAA was compared to low pH SM-II across the 'Virustatic- versus Virucidal- assay' to elucidate the pH change across the washing out of the acidity and its impact on the bacteriophage inhibition. Similarly to the previous variation, phage volumes were replaced with phage-free SM-II in the pre-dilution aliquot stage, and bacteria-free LB was used for the dilutions. Pure LB was also included as a control for this assay, as the pH of the media varied slightly between stocks. Both assay variations were carried out as biological singlets calibrating the pH meter before each assay.

Supplementary Data.

Low-throughput testing of commercial PAA.

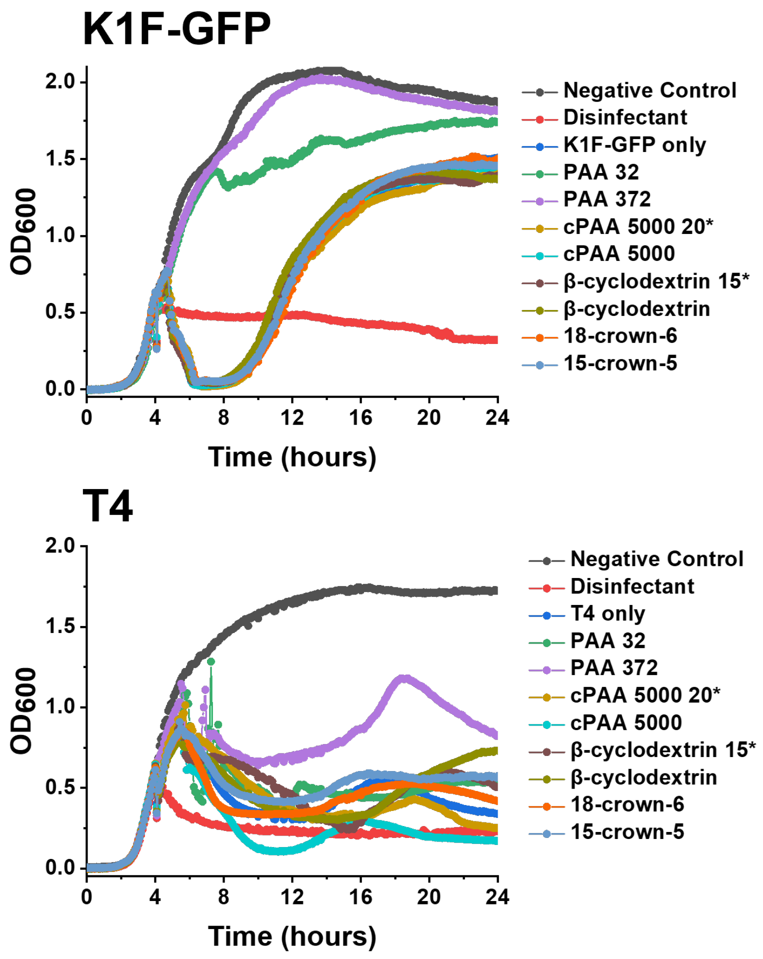

Figure S1. Low-throughput commercial poly(acrylic acid) phage inhibition screening. Growth curves for *E. coli* targeting K1F-GFP and T4 bacteriophages, respectively. Unless marked by an asterisk (15* and 20*), the phages were incubated in 10 mg.mL^{-1} of additive for 24 hours before adding the phage/additive aliquot to the pre-log phase (4-hour grown) host cultures. *E. coli* EV36 was used as the bacteria host for the K1F-GFP phage, and *E. coli* K-12 was used as the bacteria host for T4 phages, with a starting concentration of 1×10^6 CFU.mL^{-1}. K1F-GFP and T4 'only' controls refer to non-additive-containing phage aliquots. LB media was used as the negative control, and 1% Chemgene was used as a disinfectant (positive control) for the growth curves. cPAA 5000 = commercial PAA (Mw 5000). The growth curves represent one biological and two technical replicates.

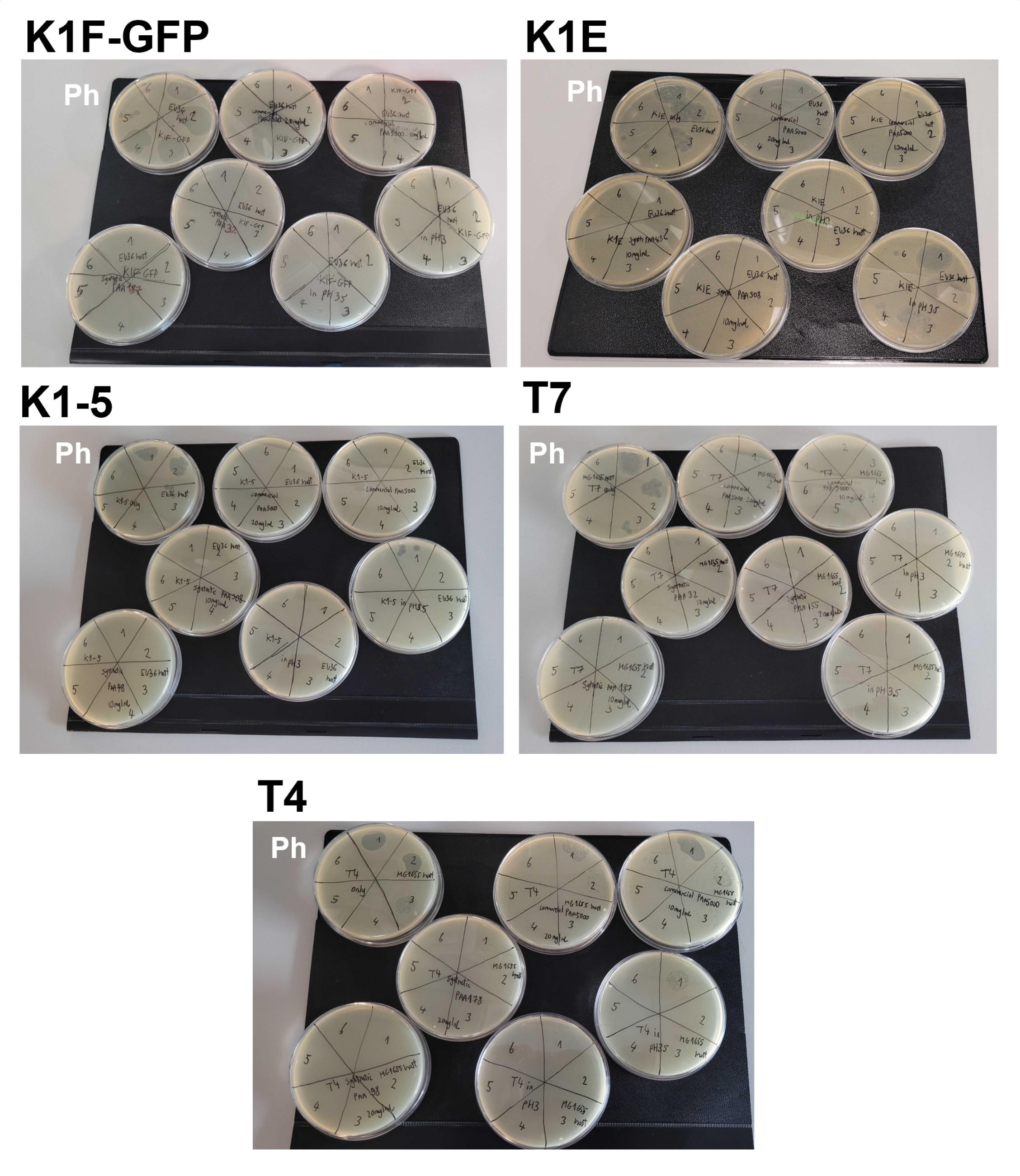

Figure S2. Photographs of each bacteriophage spot test. *E. coli* EV36 was used as the bacteria host for K1F-GFP, K1E, and K1-5 phages, whilst *E. coli* K-12 (MG1655 cells) was used as the bacteria host for T7 and T4 phages. Commercial PAA was acidified to pH 3. The polymer concentration was 10 mg.mL^{-1} unless labelled on the plates. Except for T4, all phages were incubated in 10 mg.mL^{-1} PAA 98 and PAA 178. ‘Ph’ notation in the spot-test photographs (top left of each) represents the fresh phage plates (negative control).

Low pH dose-response growth curves.

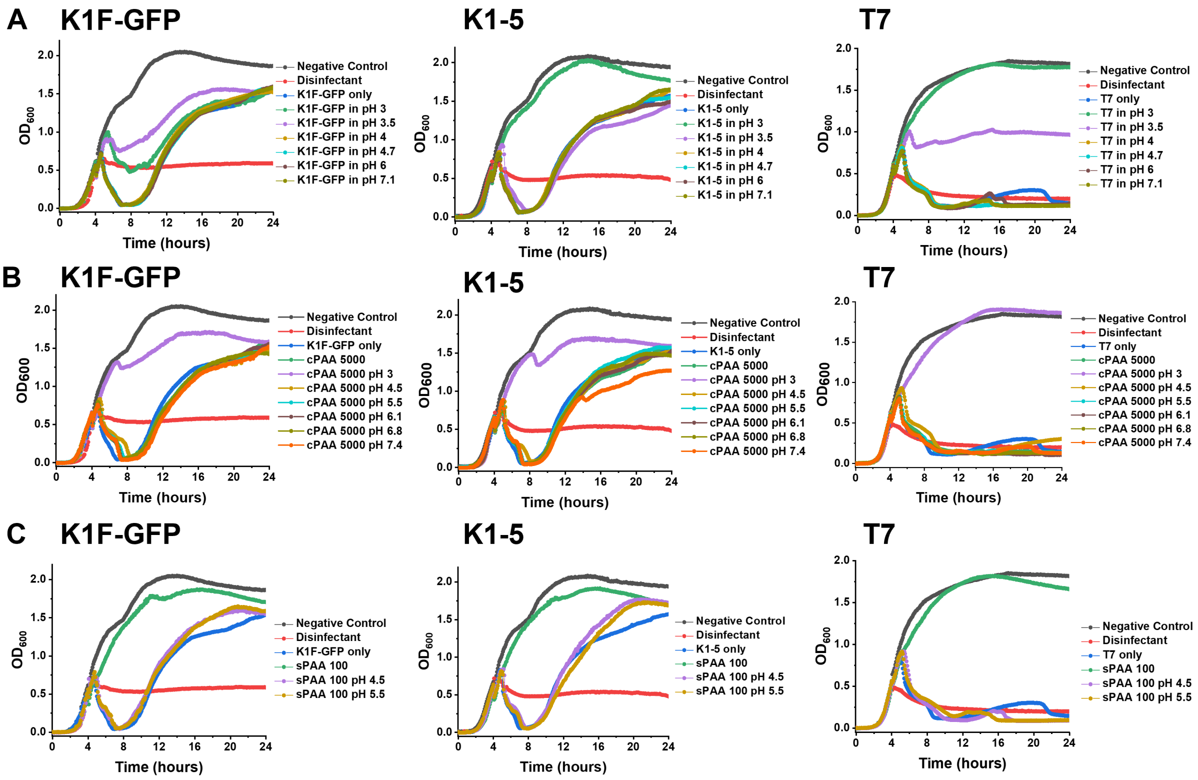

Figure S3. Low pH inhibition dose-response growth curves of bacteriophages and host. 24-hour growth curves of K1F-GFP, K1-5, and T7 phages in three different conditions of inhibition (A) acidified SM-II buffer without PAA; (B) acidified commercial PAA (cPAA 5000); (C) basified synthetic PAA 153 (sPAA 153). *E. coli* EV36 was used as the bacteria host for K1F-GFP and K1-5 phages, whereas *E. coli* K-12 (MG1655 cells) was used as the bacteria host for T7 phages with a starting concentration of 1×10^6 CFU.mL^{-1}. The pH of the SM-II buffers with and without PAA were modified before incubating with the bacteriophages. Where applicable, additive concentration was 10 mg.mL^{-1} before incubating for 24 hours and adding to relevant log phase (4 hours) host cultures Phage-only samples correspond to non-additive containing bacteriophage aliquots, LB media was used as the negative control, and 1% Chemgene as a disinfectant. The growth curves represent one biological repeat and technical triplicates.

pH tracking assays.

Table S1. pH change tracking for PAA and PMA across the growth curve assay.

Condition	PAA 98 pH	PMA 155 pH
20 $mg.mL^{-1}$ aliquot	3.02	3.07
10 $mg.mL^{-1}$ aliquot	3.49	3.8
Pseudo phage aliquot*	3.57	3.94
Pseudo phage + host (in LB)**	5.38	6.65
Pure LB	8.09	8.09

*Pseudo phage aliquot = SM-II buffer + polymer with the same final polymer concentration as the phage/polymer aliquots previously incubated for 24 hours before testing

**Pseudo phage + host (in LB) = 10-fold diluted 'Pseudo phage aliquot' in LB (no bacteria) to match the addition of phages to growing host cultures at the 4-hour pre-log phase.

Table S2. pH change tracking for PAA and acidified SM-II across washing.

Condition	PAA 98 pH	Acidified SM-II pH
Pseudo phage aliquot	3.66	2.75
Pseudo phage + host (in LB)	5.44	6.55
1st wash	6.61	6.75
2nd wash	6.78	6.78
3rd wash	6.82	6.79
Pure LB	6.70	6.70

References

(1) Richards, S.-J.; Gibson, M. I. Optimization of the Polymer Coating for Glycosylated Gold Nanoparticle Biosensors to Ensure Stability and Rapid Optical Readouts. *ACS Macro Lett.* **2014**, *3* (10), 1004–1008.

(2) Marton, H. L.; Kilbride, P.; Ahmad, A.; Sagona, A. P.; Gibson, M. I. Anionic Synthetic Polymers Prevent Bacteriophage Infection. *J. Am. Chem. Soc.* **2023**, *145* (16), 8794–8799.

(3) Cooper, P. D. The Plaque Assay of Animal Viruses; 1962; pp 319–378.
